# Overexpression of transforming growth factor type III receptor restores TGF-β1 sensitivity in human tongue squamous cell carcinoma cells

**DOI:** 10.1042/BSR20150141

**Published:** 2015-08-28

**Authors:** Duo Li, Dongyang Xu, Zhiyong Lu, Xingli Dong, Xiaofeng Wang

**Affiliations:** * Department of Oral and Maxillofacial Surgery, the Second Affiliated Hospital of Harbin Medical University, Harbin 150081, P. R. China; † Department of Biochemistry and Molecular Biology, Harbin Medical University, Harbin 150081, P. R. China

**Keywords:** apoptosis, cell cycle, tongue squamous cell carcinoma, transforming growth factor type III receptor (TβRIII), transforming growth factor-β1 (TGF-β1)

## Abstract

The transforming growth factor type III receptor (TβRIII), also known as β-glycan, is a multi-functional sensor that regulates growth, migration and apoptosis in most cancer cells. We hereby investigated the expression of TβRIII in clinical specimens of tongue squamous cell carcinoma (TSCC) and the underlying mechanism that TβRIII inhibits the growth of CAL-27 human oral squamous cells. The TSCC tissues showed a significant decrease in TβRIII protein expression as detected by immunohistochemistry (IHC) and western blot analysis. Transfection of TβRIII-containing plasmid DNA dramatically promoted TGF-β1 (10 ng/ml)-induced decrease in cell viability, apoptosis and cell arrest at the G_0_-/G_1_-phase. Moreover, transient overexpression of TβRIII enhanced the TGF-β1-induced cyclin-dependent kinase inhibitor 2b (CDKN2b) and p38 protein activity, but did not affect the activities of extracellular signal-regulated kinase 1/2 (ERK1/2) and c-Jun N-terminal kinase 1/2 (JNK1/2) in CAL-27 cells. These results suggest overexpression of TβRIII receptor restored TGF-β1 sensitivity in CAL-27 cells, which may provide some new insights on exploiting this molecule therapeutically.

## Abstract (precis)

Down-expression of transforming growth factor type III receptor (TβRIII) in tong squamous cell carcinoma clinical specimens. Overexpression of TβRIII restores TGF-β1 sensitivity in human CAL-27 tong squamous cell carcinoma cell. TβRIII over-expression affects TGF-β1-mediated activation of p38 and CDKN2b in CAL-27 cells.

## INTRODUCTION

Human tongue squamous cell carcinoma (TSCC) is the most common type of oral cancer in South Asia [1]. In the past several decades, TSCC has also been identified as the major cause of head and neck cancer-related deaths due to its frequent metastasis and poor treatment outcomes [2,3]. The 5-year survival rate of patients with TSCC ranges from 40% to 60% [4]. To improve the clinical outcome of TSCC, a better understanding of the molecular mechanism of this malignancy is required.

Transforming growth factor-β (TGF-β) initially acts as a tumour suppressor in the process of oncogenesis by inhibiting cell proliferation, maintaining tissue architecture, decreasing genomic instability and inducing cell arrest and apoptosis [5–7]. However, at later stages, cancer cells become highly resistant to the anti-proliferative effects of TGF-β and TGF-β signalling then switches to promote cancer progression [8]. Therefore, it is compelling to identify novel regulators that are closely related to TGF-β signal transduction, as well as those that promote sensitivity to TGF-β mediated anti-tumour effects. TGF-β has several types of receptors. Among them, the transforming growth factor type III receptor (TβRIII), also known as β-glycan, is the most abundant TGF-β receptor in many different cell types [9,10]. As a TGF-β superfamily co-receptor, the most well characterized role for TβRIII is to present ligands to the TGF-β signalling pathway [11].

In most patients with cancer, the expression of TβRIII is significantly decreased and the protein then functions as a suppressor of cancer progression in kidney, pancreatic, prostate, lung, breast and ovarian carcinomas [9,11,12]. Meng et al. [13] previously reported that TβRIII expression in patients with oral squamous cell carcinoma (OSCC) is down-regulated, although its role remains largely unknown [13]. In the present study, we demonstrated that the expression of TβRIII is significantly decreased in TSCC patients. Transient transfection of TβRIII dramatically improved the TGF-β1-induced anti-tumour effect on human CAL-27 TSCC cells, which is associated with significant activation of mitogen-activated protein kinase (MAPK)-dependent pro-apoptotic signalling pathways and cyclin-dependent kinase inhibitor 2b (CDKN2b)-mediated G_0_/G_1_ cell cycle arrest.

## MATERIALS AND METHODS

### Clinical TSCC specimens

Patient tissue samples were obtained from the Department of Oral and Maxillofacial Surgery of the Second Affiliated Hospital of Harbin Medical University. All TSCC patients from whom tissue specimens were obtained were not treated before primary surgery. Tissue samples were snap-frozen in the operating room immediately after excision and then sent to the pathology department for diagnosis by a board-certified head and neck pathologist. The Institutional Review Board of Harbin Medical University Hospital approved the protocol used in the present study, which had been conducted in accordance with The Code of Ethics of the World Medical Association (Declaration of Helsinki) and informed consent was obtained from all participating patients or their guardians.

### Reagents

The pc-DNA3.1-mTβRIII plasmid was purchased from GeneChem Co.. Primary antibodies for TβRIII, total or phospho-extracellular signal-regulated kinases (ERKs), phospho-c-Jun N-terminal kinases (JNKs), phospho-p38 MAPK and CDKN2b (rabbit anti-human) were procured from Cell Signaling. Other chemicals were purchased from Sigma.

### Immunohistochemistry

Serial sections (5–6-μm thick) were prepared from paraffin-embedded tissue blocks and mounted on silane-coated glass slides bought from Matsunami Glass. One section from each tissue block was stained with haematoxylin and eosin (H&E), whereas the others were used for immunohistochemistry (IHC). IHC staining was performed using the standard streptavidin–biotin–peroxidase complex method. Briefly, paraffin sections of TSCC tissues were deparaffinized, heat-treated for antigen retrieval, blocked with 10% normal goat serum for 10 min and incubated with anti-TβRIII overnight at 4°C. The tissue section was then incubated with biotinylated goat anti-rabbit immunoglobulin at a dilution of 1:75 and incubated at 37°C for 30 min. Finally chromogenic reaction was developed with 3,3′-diaminobenzidine (DAB) and counterstained with haematoxylin. TβRIII expression was then assessed by two independent investigators who were blinded to the clinicopathologic data.

### Cell culture

CAL-27, a human tongue squamous cell line, was provided by Harbin Medical University and cultured in Dulbecco's modified Eagle's medium (DMEM) bought from Gibco. All cells were supplemented with 10% FBS and maintained at 37°C in an incubator containing humidified air with 5% (v/v) CO_2_.

### Cell transfection and treatment with the human TGF-β1 cytokine

Cells were transfected with pc-DNA3.1-mTGFBR3 plasmid (GeneChem Co.) at a DNA concentration of 0.5 or 1 μg/ml respectively. The pc-DNA3.1-plasmid (Shanghai GeneChem Co.) was used as an empty vector and Lipofectamine® 2000 was used as the transfection reagent. After cell transfection for 24 h, the cells were then washed twice with PBS and cultured in serum-free medium containing 10 ng/ml of TGF-β1 for 72 h.

### MTT assay

Cells were seeded on to a 96-well plate with the same starting cell density per well (2.5×10^4^ cells/well) and allowed to adhere to the bottom of each well for 24 h. After treatment, the medium in each well was removed and replaced with a PBS solution containing 5 mg/ml of MTT and then the plate was further incubated at 37°C for 3 h. All the remaining supernatant was then removed and 100 μl of DMSO was added to each well and mixed thoroughly to dissolve the formazan crystals that developed. After 10 min of incubation to ensure that all formazan crystals were dissolved, cell viability was determined by measuring the absorbance of each well at a wavelength of 570 nm. Relative cell viability was calculated by the absorbance percentage of the treatment group to that of the control group.

### Terminal deoxynucleotidyl transferase-mediated dUTP nick end-labeling (TUNEL) staining

Apoptotic cells were detected *in situ* with a Cell Death Detection Kit (Roche Diagnostics GmbH, Roche Applied Science). Peroxidase (POD) analysis was performed using a commercially available kit for immunohistochemical detection and quantification of apoptosis (programmed cell death) at the single-cell level based on labelling of DNA strand breaks, according to the manufacturer's instructions. After staining, the coverslips were examined under a confocal microscope (Olympus, FV-100). The ratio of apoptotic (TUNEL-positive) cells to total (DAPI-stained nuclei) cells was calculated (*n*=5). Measurements were performed using the Scion Image software (Beta 4.03; Scion Corporation). All of the measurements were performed in a double-blind manner by two independent researchers.

### Assessment of transmission electron microscope

To perform transmission electron microscope (TEM) analysis, the CAL-27 cells were fixed in 2.5% glutaraldehyde in PBS for 2 h at 4°C and then post-fixed in 1% osmium tetroxide. After dehydration across a series of graded ethanol baths (30%–100%) and in propylene oxide, the cells were embedded in Epon resin. Cell sections (80–200-nm-thick) were prepared using a Reichert Ultracut E microtome and stained with uranyl acetate. Grids were examined under a Jeol 1200 EXII electron microscope.

### Cell cycle analyses

The effect of TβRIII transfection on TGF-β1-induced cell cycle arrest was determined by flow cytometric analysis of DNA content of nuclei of cells following propidium iodide (PI) staining. CAL-27 cells (1×10^6^ cells) were seeded into 75-cm^2^ flasks and allowed to adhere to the culture vessel overnight. The cells were washed with PBS and fixed in 70% ethanol overnight at 4°C. The cells were then treated with 80 mg/ml RNaseA and 50 mg/ml PI for 30 min and then analysed on a Coulter Epics XL Flow Cytometer (Beckman Coulter).

### Quantification of TβRIII mRNA level

Total RNA isolated by using the TRIzol™ Reagent (Invitrogen) was treated with a Turbo DNA-free kit (Ambion) to eliminate genomic DNA contamination. The TβRIII mRNA was detected with a specific forward primer (5′-ACTGGATGAGAGGCACTG-3′) and reverse primer (5′-TGGTCCCTGTGTTTGTC-3′). The ΔCt (target–reference) was calculated for each sample. The fold change in expression of TβRIII was calculated by using the 2-ΔΔCt method, in which ΔΔCT=ΔCT (target–reference)–ΔCT (target–reference). Quantitative reverse transcription-PCR (qRT-PCR) was conducted in triplicate for each sample and an average 2-ΔΔCt value along with its S.D. was calculated for each sample relative to that of the normal control. β-Actin was used as the internal reference gene to which the expression of TβRIII was normalized.

### Western blot analysis

Cells and tissue samples were harvested and lysed in Radio-immunoprecipitation assay buffer (RIPA buffer) with protease inhibitors. Lysates (100 μg/lane) were loaded on to a 10% gradient gel for SDS/PAGE. After SDS/PAGE, the gels were blotted on to an Immunobilon-P nylon membrane. After blocking, the blots were incubated overnight with the appropriate primary antibodies against TβRIII, total or phospho-ERKs(1:500 dilution), phospho-JNKs(1:500 dilution), phospho-p38 MAPK(1:500 dilution) and CDKN2b (1:1,000 dilution), separately at 4°C overnight. Following incubation with peroxidase-linked secondary antibodies, the proteins were visualized by using an ECL reagent. Western blot bands were quantified using the Odyssey v1.2 software, measuring the band intensity (area × *D*) for each group and normalizing these to the intensity of β-actin (anti-β-actin antibody procured from Kangcheng), which acted as an internal control for the western blot assay.

### Statistical analysis

Data were expressed as the mean ± S.D. Statistical comparisons were performed with the Student's *t* test and ANOVA and differences with *P*<0.05 were considered significant.

## RESULTS

### Down-regulation of TβRIII in TSCC clinical specimens

Table 1 summarizes the clinicopathologic features of seven patients with TSCC, who provided 14 oral lesions and adjacent normal tongue tissues. All tumours were sporadic. Of these, four occurred in women and the median age at the time of presentation was 55.7 years (range: 47–72 years). The histological type of these tumours was squamous cell carcinoma.

**Table 1:** Clinicopathologic features of the TSCC samples in the present study

Case	Gender	Age (years)	Tumour location	Histological type
1	F	53	Tongue	Squamous cell carcinoma
2	F	66	Tongue	Squamous cell carcinoma
3	F	48	Tongue	Squamous cell carcinoma
4	F	72	Tongue	Squamous cell carcinoma
5	M	53	Tongue	Squamous cell carcinoma
6	M	51	Tongue	Squamous cell carcinoma
7	M	47	Tongue	Squamous cell carcinoma

**Abbreviations:** F, female; M, male.

To determine whether TβRIII might be associated with the progression of TSCC disease, an analysis of the expression of TβRIII was conducted by immunohistochemical staining. Figure 1(A) shows matched images of a normal tongue and TSCC that were stained with H&E (magnification: 40×). The tongue epithelium includes the basal layer, spinous layer, granular layer and stratum corneum. Strong immunoreactivity to TβRIII was observed in the basal layer and the upper spinous layer in normal tongue tissues (Figure 1A, upper panel, IHC, magnification: 40×, 400×, indicated by red arrows). In the lower panels, we observed carcinomatous cell nests in TSCC (Figure 1A, lower panel, yellow arrows indicate carcinomatous epithelial nests of TSCC). Carcinomatous cell nests are formed by invasion of epithelial cells into the underlying connective tissue. The TSCC cells showed weak reactivity to TβRIII and the number of TβRIII-positive cells were markedly reduced (Figure 1A, lower panel, IHC, magnification: 40×, 400×). Moreover, we also evaluated the expression levels of TβRIII protein and mRNA in TSCC clinical specimens by western blot and RT-PCR analyses. Consistent with a previous study [13], we observed a significantly lower level of TβRIII mRNA (Figure 1B) and protein (Figure 1C) in TSCC specimens compared with that of non-tumour tissues. On the contrary, western blot analysis revealed a marked increase in the level of TGF-β1 protein expression in TSCC clinical specimens (Figure 1D).

**Figure 1:**
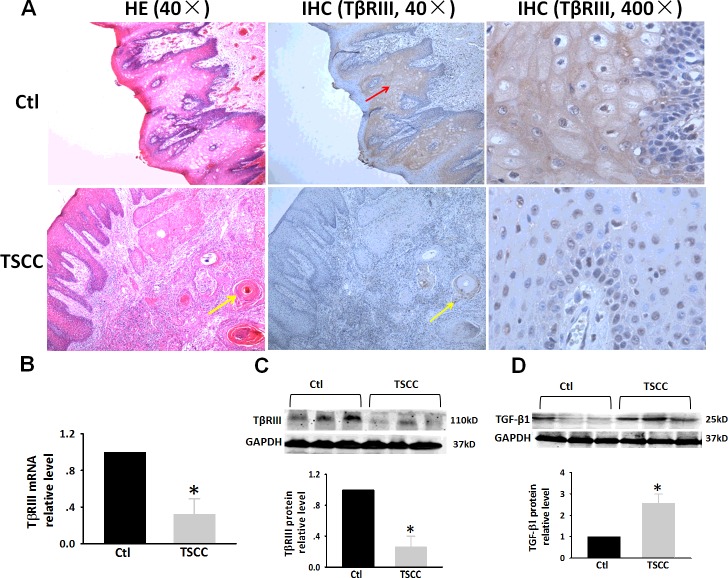
Expression of TβRIII in TSCC tissues (**A**) H&E stain and IHC analysis of TβRIII expression in matched normal human tongue tissue and TSCC specimens. (**B**) Expression of TβRIII mRNA in specimens was evaluated by RT-PCR. (**C**) Expression of TβRIII protein in specimens was evaluated by western blot analysis. (**D**) Expression of TGF-β1 protein in specimens was evaluated by western blot. Glyceraldehyde 3-phosphate dehydrogenase (GAPDH) was used as loading control. These changes were quantified using densitometry in (**C** and **D**). Data are expressed as the mean ± S.D. from three independent experiments (*n*=3). *P<0.05 compared with control.

### TβRIII overexpression sensitizes the TGF-β1-induced cell death in CAL-27 cells

To verify the role of TβRIII in the TGF-β1 signalling pathway in TSCC, we overexpressed TβRIII in CAL-27 cells (Figure 2A). Western blotting showed that the expression of TβRIII in cells treated with 0.5 μg/ml (2.31-fold) and 1 μg/ml (3.69-fold) of TβRIII plasmid DNA increased in a concentration-dependent manner. Cell viability was determined by MTT assays, as described in ‘Materials and Methods’ section. As illustrated in Figure 2(B), under control conditions, the relative viability of the CAL-27 cells was ∼100%. In the cells treated with TGF-β1 at doses of up to 10 ng/ml for 72 h, an apparently slight decrease in cell viability was observed. However, the transfection of TβRIII markedly enhanced the decrease in TGF-β1-induced cell viability by 38.8±14.1 and 48.1±12.1% respectively.

**Figure 2:**
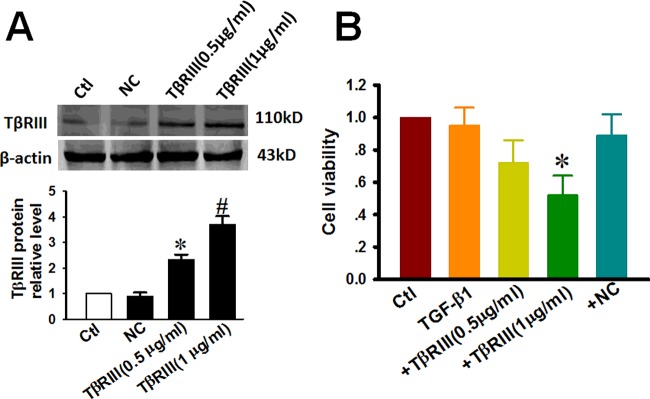
Overexpression of TβRIII contributes to the decrease in the viability of CAL-27 cells by TGF-β1 treatment CAL-27 cells were transfected with plasmid encoding TβRIII at concentrations of 0.5 μg/ml and 1 μg/ml. NC represents empty vectors (1 μg/ml of pc-DNA3.1 plasmid) transfected into CAL-27 cells, which serves as an NC. After TβRIII overexpression for 24 h, CAL-27 cells were further incubated with 10 ng/ml TGF-β1 for 72 h. (**A**) TβRIII expression as determined by western blot analysis and the averaged band intensities from three independent experiments are presented. (**B**) Relative cell viability as determined by MTT assay. Data is presented as the rate (%) of growth control (viability of control) cultured in growth medium without transfection. Abbreviations: Ctl, control; +NC, empty vector transfection + TGF-β1 48 h; +TβRIII, TβRIII transfection + TGF-β1 48 h. Data are expressed as the mean ± S.D. from three independent experiments (*n*=3). *P<0.05 compared with Ctl.

We next investigated whether or not TβRIII overexpression could improve sensitivity to TGF-β1-induced cells apoptosis in CAL-27 cells. TUNEL staining (Figures 3A and 3B) indicated that under control conditions, it was difficult to detect nuclear staining in CAL-27, whereas TGF-β1 (10 ng/ml) induced a modestly higher number of apoptotic cells. In contrast, CAL-27 cells transfected with TβRIII DNA plasmid after treatment with TGF-β1 (10 ng/mL) for 72 h showed a significantly higher percentage of stained nuclei, which was indicative of apoptosis. We also examined the effect of TβRIII overexpression on the micromorphological changes induced by TGF-β1 by EM at an original magnification of 8000×, as an alternative indication of apoptosis. As shown in Figure 3(C), the ultrastructural organization of the cells under control conditions was apparently normal. However, in the cells treated with TGF-β1, a considerable number of cells exhibiting early nuclear morphological changes during apoptosis, including the disappearance of microvilli and chromosomal DNA condensation, were observed. Furthermore, some of the late-stage apoptotic changes were observed in TGF-β1-treated cells transfected with TβRIII DNA plasmid such as mitochondrial swelling and formation of separated apoptotic bodies (Figure 3C), white arrows indicate mitochondrial swelling and red arrows indicate apoptotic bodies). Moreover, the effect of TβRIII overexpression on cell cycle distribution provided insights into the mechanism underlying its anti-proliferative activity. Figure 3(D) shows that exposure of CAL-27 cells to TGF-β1 for 72 h resulted in the overexpression of TβRIII, which in turn led to the significant accumulation of cells in G_0_/G_1_-phase, coupled with a decrease in the number of cells at the G_2_/M transition. For example, compared with the TGF-β1 alone group that was treated for 72 h, the percentage of cells in G_0_/G_1_-phase increased by 2.2-fold upon TβRIII overexpression (Figure 3D).

**Figure 3:**
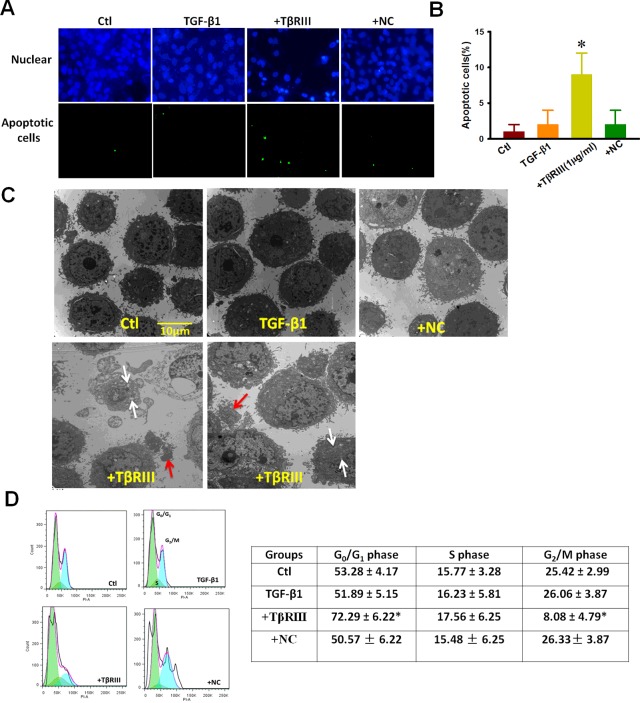
TβRIII overexpression promotes apoptosis and G_0_/G_1_ arrest in CAL-27 cells by TGF-β1 treatment After TβRIII overexpression for 24 h, CAL-27 cells were further incubated with 10 ng/ml of TGF-β1 for 72 h. (**A**) TUNEL staining assays. (**B**) Percentage of TUNEL-positive cells obtained from different experimental conditions (**C**) TEM images showed micromorphological changes (magnification: 8000×; white arrows indicate mitochondrial swelling and red arrows indicate apoptotic bodies). (**D**) Analysis of cell cycle distribution by flow cytometry. Abbreviations: Ctl, control; +NC, empty vector transfection +TGF-β1 48 h; +TβRIII, TβRIII transfection + TGF-β1 48 h. Data were obtained from four experiments (*n*=4). Values are expressed as the mean±S.D. *P<0.05 compared with Ctl.

### TβRIII overexpression affects TGF-β1-mediated activation of p38 and CDKN2b in CAL-27 cells

MAPKs, including ERK, JNK and p38, are a family of downstream signalling molecules that respond to TGF-β1 stimuli and are known to be involved in cell survival and death decisions. We hypothesized that TβRIII promotes TGF-β1-induced apoptosis in CAL-27 cells by enhancing the activity of these kinases. To this end, we performed western blot analysis with respect to the expression levels of these kinases. As illustrated in Figure 4, cells treated with 10 ng/ml TGF-β1 for 1 h in the presence of TβRIII transfection resulted in a significant increase in total and phosphorylated p38 MAPK activities (Figure 4C), whereas the activity of ERK and JNK did not change (Figures 4A and 4B). These results suggest that p38 MAPK-dependent signalling might play a role in the TβRIII-enhanced increase in sensitivity to TGF-β1-induced apoptosis in cultured CAL-27 cells. To elucidate the mechanism underlying the G_2_/M arrest in TβRIII-affected CAL-27 cells, its effect on the expression of CDKN2b that regulates the G_0_/G_1_ transition was determined by western blot analysis and representative blots are shown in Figure 4(D). After the cells were treated with 10 ng/ml TGF-β1 for 24 h, western blotting revealed that TβRIII transfection resulted in a significant increase in the protein levels of TGF-β1-activated CDKN2b in a time-dependent manner. These results suggest that TβRIII transfection increases the kinase activity of CDKN2b to promote G_0_/G_1_ phase cell cycle arrest.

**Figure 4:**
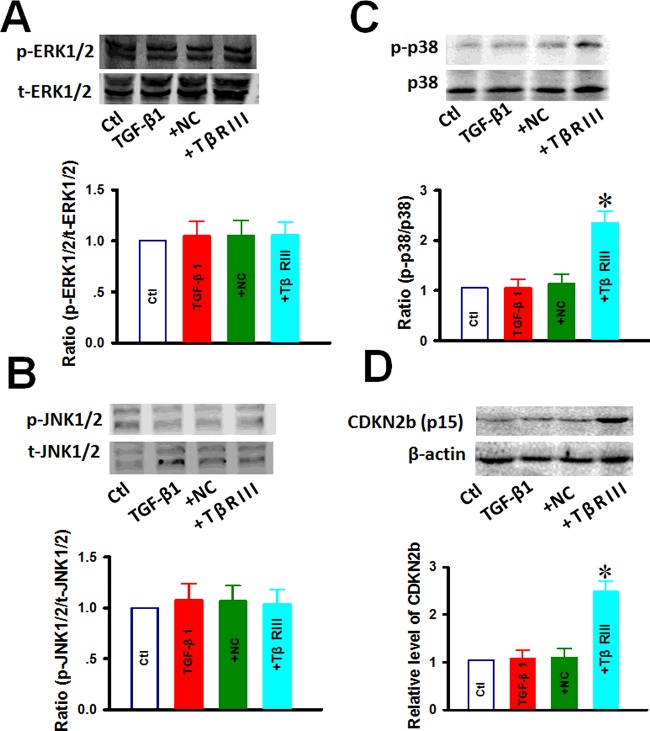
Effect of TβRIII overexpression on CDKN2b and MAPKs proteins of CAL-27 cells After TβRIII overexpression for 24 h, CAL-27 cells were further incubated with 10 ng/ml of TGF-β1 for 1 h or 24 h. Western blotting was used to detect ERK (**A**), JNK protein (**B**), p38 (**C**) and CDKN2b (**D**). Abbreviations: Ctl, control; +NC, empty vector transfection + TGF-β1 1 h; +TβRIII, TβRIII transfection + TGF-β1 1 h. Data were obtained from four experiments (*n*=4). Values are expressed as the mean ± S.D. *P<0.05 compared with Ctl.

## DISCUSSION

The present study showed that the level of expression of TβRIII decreased in TSCC and this may facilitate our better understanding of the dual tumour suppressor/tumour promoter roles of the TGF-β in early- and late-stage human cancers. We also identified a novel cellular function of TβRIII, namely, to enhance the anti-proliferative effects of TGF-β signalling in CAL-27 cells by promoting apoptosis and cell arrest at the G_0_/G_2_ phase. We further elucidated that increasing the activation rate of both p-38 and CDKN2b serves as an important mechanism for TGF-β1-induced apoptosis and cell arrest, thereby suppressing TSCC cancer cell growth.

Loss of growth inhibition by TGF-β is a common feature of tumour cells and its expression and large secretion is elevated in most cancer cells [9,11]. Similarly, Nair et al. [14] reported that TGF-β1 expression is significantly increased in clinical specimens with OSCC. On the contrary, an expanding body of evidence indicates that a dysfunctional TβRIII mechanism due to loss of TβRIII receptors leads to the development and progression of several human malignancies, including breast cancer, prostate cancer and head and neck squamous carcinoma [12,13,15,16]. TβRIII expression is negatively regulated at the transcriptional level by TGF-β1 through the inhibition of the proximal promoter in multiple cell types, including breast, ovarian and small cell lung cancer cell lines [17,18]. Consistently, the present results suggest that the decrease in TβRIII mRNA and protein expression in human TSCC cancer cells might be possibly due to TGF-β1 overexpression, which are resistant to the negative growth effects of TGF-β1 due to the loss of TβRIII, thereby restoring TGF-β1 sensitivity. In general, TβRIII can inhibit or enhance the signalling of particular TGF-β superfamily members via cell type/context-dependent mechanisms that are still poorly understood. In the present study, we demonstrated that gene transfer-mediated overexpression of TβRIII in CAL-27 TSCC cancer cells restored TGF-β1 sensitivity and inhibited their tumorigenic behaviour. These results indicate that CAL-27 cells are refractory to TGF-β1 negative growth effects due to the lack of TβRIII expression.

Several efforts have been made to examine the effect of TβRIII on cell viability and apoptosis in different cell types [19,20]. For instance, Zheng et al. [19] demonstrated that transient overexpression of TβRIII enhanced caspase-3 activity and induced apoptosis in human nasopharyngeal carcinoma CNE-2Z cells without TGF-β stimulation. Moreover, a study conducted by Chu et al. [20] also revealed that overexpression of TβRIII protects cardiac fibroblasts from hypoxia-induced apoptosis. Interestingly, overexpression of TβRIII had no significant effect on the rate of MDA-MB231 breast cells division, nor did it restore cell responsiveness to TGF-β-induced growth inhibition [12]. However, it markedly inhibited TGF-β-induced invasion and significantly attenuated the responsiveness of the MDA-MB231 cells to TGF-β-induced Smad activation [12].

It appears that the overexpression of TβRIII-induced decrease or increase in cell viability largely depends on the particular cell type. In general, TβRIII exerts its effect by directly presenting the TGF-β ligand to TβRII and by subsequently recruiting TβRI for downstream cytoplasmic signalling via multiple parallel signalling pathways [9]. In the present study, our results indicated that the overexpression of TβRIII in CAL-27 TSCC cells caused tumour apoptosis and cell arrest by restoring TGF-β1 activity in multiple signalling pathways. Previously, studies have indicated that TβRIII also regulates the p38 signalling pathway in various cell types [21–23]. For instance, in L6 myoblasts, the presence of TβRIII results in the activation of the p38 MAPK pathway in a TβRI receptor-dependent manner [21]. However, in a renal cancer cell line, TβRIII expression similarly resulted in p38 activation, although in a TβRII receptor-independent manner [22]. Consistent with our results, increasing TβRIII expression in colon cancer model systems enhances TGF-β-mediated phosphorylation of p38, whereas ERK signalling does not change [23]. Sun et al. [10] reported that TβRIII inhibits ERK1/2 and JNK signalling by interacting with GAIP-interacting protein C-terminus (GIPC) in neonatal mouse cardiac fibroblasts. On the other hand, our results showed that the activity of ERK and JNK did not change with TβRIII transfection of CAL-27 cells. It is also possible that CDKN2b is involved in the overexpression of TβRIII that accelerated CAL-27 cells arrest at the G_0_/G_1_-phase induced by TGF-β1 treatment. The results described in the present study are consistent with several earlier investigations. For instance, Robson et al. [24] discovered that TGF-β1 is inhibitory to most epithelia, which are caused by the up-regulation of CDKN2b and a delay in the cell cycle at the G_1_-phase [24]. Wu et al. [25] demonstrated that the autocrine TGF-β/CdKN2b signalling mechanism of podocytes specifies the G_0_/G_1_ arrest associated with podocyte differentiation and TGFβ-induced apoptosis associated with selective p38 mitogen-activated protein kinase activation. Liu et al. [26] revealed that TGF-β1 inhibits cell proliferation in the OSCC brain metastasis Tb cell line and blocks the cell cycle at the G_1_-phase via increasing the expression of CDKN2b.

In conclusion, we have demonstrated that overexpression of the *TβRIII* gene in the TGF-β-resistant tongue cancer cell line, CAL-27, increases its sensitivity to the growth inhibitory effect of TGF-β by restoring functionality of the TGF-β1 signalling pathway. These observations support the role of TβRIII as a potential tumour suppressor gene in TSCC. Restoration of the expression of TβRIII in the TSCC cells may serve as a novel approach for the treatment of oral carcinoma.

## Abbreviations

H&E: haematoxylin and eosin
IHC: immunohistochemistry
OSCC: oral squamous cell carcinoma
PI: propidium iodide
TGF-β: transforming growth factor-β
TSCC: tongue squamous cell carcinoma
TβRIII: transforming growth factor type III receptor
